# A review on PIM kinases in tumors

**DOI:** 10.6026/97320630015040

**Published:** 2019-02-03

**Authors:** Housna Arrouchi, Wiame Lakhlili, Azeddine Ibrahimi

**Affiliations:** 1 Laboratory of Biotechnology (MedBiotech),Rabat Medical and Pharmacy School,Mohammed V University in Rabat, Rabat,Morocco

**Keywords:** Tumor, Pim-1 kinase, Small molecule inhibitors

## Abstract

The Proviral Integration site for Moloney murine leukemia virus (PIM) kinases is serine/threonine kinases that promote growth and survival in multiple cell types, implicated in the pathogenesis of various diseases. Over expression of Pim-1 experimentally leads to tumor formation in mice, whereas there is no observable phenotype concerning the complete knockout of the protein. When it is over expressed it may lead to cancer development by three major ways; by inhibiting apoptosis, by promoting cell proliferation and also through promoting genomic instability. Expression in normal tissues is nearly undetectable. Recent improvements in the development of novel inhibitors of PIMs have been reviewed. Significant progress in the design of PIMs inhibitors, in which it displays selectivity versus other kinases, has been achieved within the last years. However, the development of isoform-selective PIM inhibitors is still an open task. As Pim-1 possesses oncogenic functions and is over expressed in various kinds of cancer diseases, its inhibition provides a new option in cancer therapy. A PubMed literature search was performed to review the currently available data on Pim-1 expression, regulation, and targets; its implication in different types of cancer and its impact on prognosis is described. Consequently, designing new inhibitors of PIMs is now a very active area of research in academic and industrial laboratories.

## Background

The human PIM kinase is a highly conserved serine-threonine protein kinase (STPK) named for the genomic site where it was discovered, Proviral Integration site for MuLV (The murine leukemia virus) [1]. The PIM kinases promote growth and survival in multiple cell types [2-3]. Composed of three isoforms: Pim-1, Pim- 2, and Pim-3, the PIM family play a critical role in the control of cell proliferation, survival [4], and migration [5-6]. Her over expression has been reported in human tumors, mainly in hematologic malignancies and in multiple other cell types, including vascular smooth muscle [7], cardiomyocytes [8], and breast [9]. They are constitutively active kinases regulated through expression and rapid turnover downstream of growth factor signaling [10]. Designing new inhibitors of PIMs is the latest advances of research in academic and industrial laboratories. In this review, our objective is to provide the role of the Pim-1 in cancer pathology and the state of the art in the design and development of PIM inhibitors. Similarly, we aim to highlight the structural elements that can discriminate between PIM isoforms to design selective inhibitors, by analyzing published data from the crystallized complexes of Pim-1 and their inhibitors, and pharmacology data, as well as from the clinical data.

## Physiological and Pathological Role of Pim-1 kinase:

Activation by other protein kinases is unneeded for Pim-1 in contrast to other STPKs, in particular, mitogen-activated protein kinases and protein kinases A, B, and C. The protein kinase is constitutively active, and its phosphorylation stabilizes the enzyme but is not essential for the regulation of its catalytic activity [11]. The PIM stability is negatively regulated by phosphatase 2A, indicating that auto phosphorylation and/or phosphorylation by still unknown partners may play an important role in regulating PIM activity [12]. Pim-1 can be mainly expressed in the thymus, spleen, bone marrow, fetal liver and other hematopoietic organs, while its expression is absent in adult tissues. Recent studies have shown that Pim-1 kinase can phosphorylate a variety of protein substrates, and it is a downstream effector molecule of many cytokine signaling pathways [2]. The pro survival function of the PIM proteins in immunology is parallel to their established role in oncology. Where by abnormal expression of these kinases, has been linked to various human cancers, including prostate [13], pancreatic [14], colon [15], chronic lymphocytic leukemia, non-Hodgkin's lymphoma, [16] and multiple myeloma [17]. Pim-1 plays an essential role in arterial wall cell proliferation and associated vascular diseases; which includes pulmonary arterial hypertension and aortic wall neo-intima formation. Moreover, Pim-1's role in high-glucose (HG)-mediated vascular smooth muscle cell proliferation has been tested [18]. It has been shown that hypoxia induces Pim-1 expression, which promotes solid tumor growth [19-20]. In addition to hypoxia and oxidative stress, anti-tumor drugs can sometimes induce stress that results in enhanced tumor survival. A mechanism underlying such a response was determined for the anticancer drug docetaxel [21].

## Pim-1 kinase as a target for cancer therapy:

Druggability of the PIM family has been established, by the development of various potent and selective chemotypes, but some common cross-reactivities have also been observed, including CK2, PI3K, and PKCe [22-23]. All three enzymes Pim-1, Pim-2, and Pim-3, lack a regulatory domain, suggesting a constitutive activity which, is directly correlated with their expression levels [24]. Among these three enzymes, the role of Pim-1 as an oncogene has been demonstrated, in a number of studies [25]. Pim-1 is aberrantly up regulated, in a variety of human cancers and both in vitro and in vivo studies have evidenced the role of Pim-1 in biological activities of cancerous cells, such proliferation, cell cycle progression, apoptosis, invasion, and glycolysis [25-26].

Over expression of PIM kinases has been observed, in hematological cancers, prostate cancer, pancreatic cancer, gastric cancer, head and neck cancer, colon cancer, and liver cancer [27]. Dysregulated expression of PIM kinases has been strongly implicated in tumorigenesis through cooperation with MYC, mediating survival signaling, and regulation of cell cycle progression [28]. In normal cells, cytokine signaling through the JAK/STAT pathway strictly regulates the transcriptional activation of the Pim genes and their expression as constitutively active protein kinases. Mainly in liquid and solid tumors [29], PIM kinases are over-expressed and this has been associated, with drug resistance mechanisms [30]. The observation that PIM kinases are selectively over-expressed in certain cancers, while apparently playing non-essential roles in normal cells, prompted numerous research groups [31-35] to initiate medicinal chemistry efforts to identify small molecule inhibitors of PIM kinases. In humans, an enhanced level of nuclear Pim-2 in the tumor cells has been associated with a higher risk of prostatespecific antigen recurrence and with peri neural invasion of the prostate gland. Consistently, over expression of Pim-1 has been reported to be related to the grade of prostate cancer [36]. In the context of Hepatocellular carcinoma (HCC), Pim-1was found overexpressed in primary-HCC tissue and positively associated with extra-hepatic metastasis. Furthermore, Pim-1 knockdown could suppress HCC proliferation and invasion in vitro and tumor growth and metastasis in vivo [37].

## Pim-1 cooperates with MYC in triple-negative breast cancer:

According to the expression condition of biomarkers, there are three types: hormone-receptor-positive human epidermal growth receptor 2 (Her-2)-positive or triple-negative breast cancer (TNBC) (known as TNBCs, defined by the lack of ER, PR, and Her-2) which contains 15% of the whole breast cancer population [38]. Many patients diagnosed with TNBCs are resistant to the therapy at the beginning of treatment, and a large number of patients cannot tolerate the side effects. The MYC oncogene was found overexpressed in TNBCs compared with other subtypes and especially in those resistant to chemotherapy, but the inhibition has been challenging to achieve [39-40]. Recently, there are two articles demonstrating that Pim-1 cooperates strongly with c-MYC and Pim-1 inhibitors can inhibit cell proliferation, migration, and apoptosis in TNBCs. The level of Pim-1 mRNA is significantly higher in TNBCs than in other subtypes [41- 42]. Based on these facts, Pim-1 may be a promising target for TNBCs [43]. Pim-1 is a coactivator of MYC, and PIM kinase phosphorylation of histone H3 at serine 10 leads to stimulation of RNA polymerase II binding, which results in increased c-Myc- driven transcription [44]. It is approximated that PIM1 is required, for the expression of 20% of total MYC target genes.

## Role of the Pim-1 kinase in therapeutic resistance:

Interestingly, some studies have demonstrated that Pim-1 plays an important role in glycolysis, which is the major source of energy for cancerous cells [45-46]. Moreover, the association between Pim-1 and drug-resistance of cancer cells has also been established. It has been found that Pim-1 mediates drug resistance through interaction with Etk, P-glycoprotein (Pgp) and there phosphorylation, breast cancer resistant protein (BCRP) and fms-like tyrosine kinase 3 (FLT3) [47]. Physcion-8-O-b-glucopyranoside (PG) significantly repressing expression of Pim-1 and changed the expression of Bcl-2 and Bcl-xL, suggesting that PG induced apoptosis in HCC cells by regulating the expression of Pim-1 and consequently modulating the effector molecules that are substrates of PIM [37,48].

## Structural Insights into PIM Inhibition:

**Figure 1:**
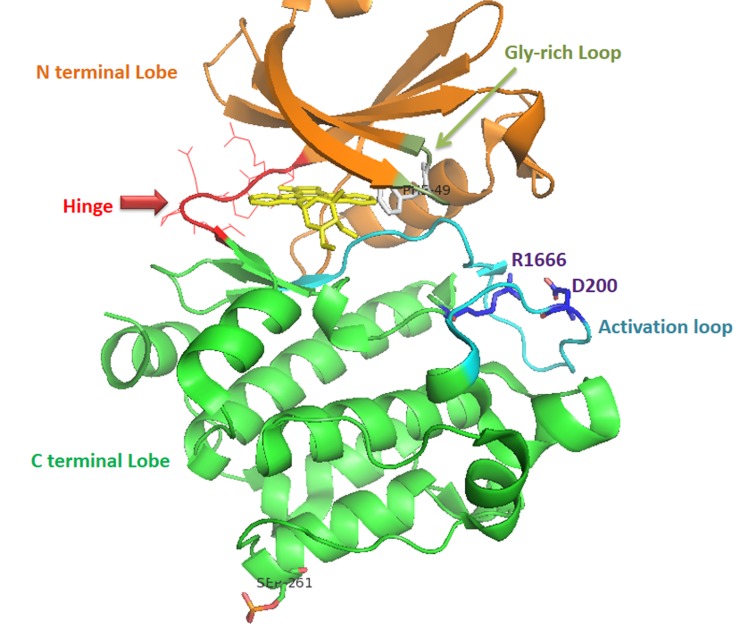
Structure of the Pim-1-staurosporine complex (ID PDB: 1YHS). The structure is shown with β-sheets as arrows and the a- helices. The N-terminal domain (colored orange) is shown with the glycine-rich loop drawn in green. The hinge connecting the two domains is colored red. The C-terminal domain is shown in green with the activation loop shown in cyan. Staurosporine (yellow) is shown in the active site, bound between the Phe49 side chain (colored gry in the glycine-rich loop) and the hinge region. The salt bridge stabilizing the conformation of the activation loop is formed by residues Asp200 and Ar166 side chains, drawn with blue carbon atoms. The site of phosphorylation, Ser361 is shown. The image was prepared with Pymol.

All three PIMs bound the natural substrate ATP via only one hinge hydrogen bond involving the adenine amino substituent and the E121 backbone carbonyl. P123 of the hinge lacked the hydrogen bond donor functionality normally present in other kinases to interact with the ATP adenine ring nitrogen [24,49]. The hinge also contained V126 insertion that was absent in other kinases. This insertion changed the hinge conformation enlarging the adjacent binding pocket. Such differences between PIMs and other kinases presented an opportunity for developing selective pan-PIM inhibitors [50-51]. The PIM kinases retain a unique consensus hinge region sequence LERPXPX (L is the gatekeeper residue) which, affords some interesting consequences, and several cocrystal structures of Pim-1 with ATP analogs or inhibitors have now been reported, demonstrating some remarkable binding features [52]. The crystal structures of Pim-1 complexed with staurosporine and adenosine [18] are shown in Figure 1. Crystallographic studies of Pim-1 have identified unique structural features but have not provided insight into how the kinase recognizes its target substrates [53]. In addition to a strong preference for basic residues, particularly arginine, at the -5 and -3 positions, the common PIM kinase motif is characterized by selectivity at a number of other sites, including histidine at the -2, proline at the -1, and glycine at the -1 position [24,53]. The consensus sequence for Pim-1 substrate recognition is Lys/Arg-Lys/Arg-Arg-Lys/Arg-Leu-Ser/ Thr-Xaa, where Xaa is an amino acid with a small side chain [54].

In the active conformation, ATP binds the hinge region by means of two hydrogen bonds, while the phosphate groups interact with two lysine residues and two magnesium ions, which in turn coordinate to Asx or Glx side-chain oxygen atoms. The presence of a proline residue at position 123 of Pim-1 makes this protein unique in comparison with other protein kinases. In fact, P123, because of its inability to act as hydrogen bond donor, prevents the formation of the canonical second hydrogen bond between the adenine moiety of ATP and the hinge backbone. In silico and in vitro approaches were used, in order to identify inhibitor scaffolds for Pim-1. We screened a small kinase targeted library of about 200 compounds and determined the Structure-Activity Relationship for commercially available analogs of the identified scaffolds [55]. The potential for the development of Pim-1-selective inhibitors is enhanced, by the crystal structure of Pim-1, which has been recently solved by multiple groups [54]. Importantly, the hinge region that contains the ATP binding site has a novel architecture, containing an additional amino acid residue not found in other protein kinases. This residue, proline-123, is incapable of making hydrogen bonds with ATP because of lack of a key amide H-bond donor. This structural feature is a key determinant in the binding mode of PIM inhibitors, [54] and several groups have reported structurally novel PIM kinase inhibitors.

## Pim-1 inhibitors:

The structural studies revealed that the Pim-1 protein has classical bilobal kinase domain architecture and, apart from a unique Nterminal peptide sequence, has all of the conserved secondary structure elements of typical protein kinases. However, the unique hinge architecture of Pim-1 kinase suggests that very selective inhibitors can be identified using this structural difference [56]. Indeed, over 50 potential Pim-1 inhibitors have been selected by Targeting Unique Structure of ATP-Binding Pocket [57], but the proteome-wide specificity of these inhibitors is largely unknown [58]. Several different classes of Pim-1 inhibitors have recently been reported, including ruthenium-containing organometallic complexes [59-60], bis-indolylmaleimides [61], imidazo[1,2-b]pyridazines [62], pyridine [63], flavonoids [64-65], benzoisoxazoles [66], isoxazoloquinoline-3,4(1H,9H)-diones [67], 5-arylidene-2,4- thiazolidinediones [68], Cinnamic acids [69] and 3Hbenzo[4,5]thieno[3,2-d]pyrimidin-4-ones [70] and with Rhodamine- Benzoimidazole structure [71]. Most Pim-1 inhibitors work either as ATP competitors or as ATP mimetic compounds (Table 1).

In addition, several inhibitors, such as isoxazoloquinoline-3,4 (1H,9H)-diones, interact via halogen atoms with the Pim-1 hinge region. Others have also been shown to impair the growth of cancer cell lines in vitro [72,73,29]. Importantly, it has been demonstrated that SGI-1776, induces apoptosis in chronic lymphocytic leukemia cells and re-sensitizes chemoresistant cancer cells to taxanes [29]. Furthermore, SGI-1776 was shown to be a fairly selective inhibitor of the PIM kinases in a broad scale kinase screen, although it is also noteworthy that SGI-1776 shows the most potent activity against Pim-1 compared with Pim-2 and Pim-3 [74]. The Representative pan-PIM inhibitors are AZD1208[40], and PIM447, the clinical trials of PIM447 are underway in phase I (NCT02370706). The effects of AZD1208 as a single agent and in combination with an Akt inhibitor were investigated, in a large panel of gastric cancer cell lines through growth inhibition assays [75].

## Conclusion

A large body of biological data suggests inhibition of Pim-1 as an interesting point of intervention to treat certain human leukemia and lymphomas. Due to the unique hinge region (characterized both by an atypical conformation and by the lack of a hydrogen bond donor at position 123) it is a particular importance to examine how Pim-1 binds small molecule inhibitors. The lack of this hydrogen bond donor in kinases of the PIM family has been reported to produce a significantly altered selectivity toward small molecules as compared to other serine/threonine protein kinases. For example, in a recent publication by Kumar et al. an unexpected binding mode for AMP and an inhibitor of the oxindole class have been described. Interestingly, for publications, which provide selectivity data comparing Pim-1 and Pim-2, selectivity for Pim-1 over Pim-2 is observed, which is surprising considering the degree of homology between these enzymes.

## Conflict of Interest

The authors declare that they have no competing interests.

## Figures and Tables

**Table 1:** Characteristics of Pim-1 inhibitors

Name of inhibitors	Mechanism of inhibition	IC50 (or Ki)
Orgametallic complexes	ATP mimetic	10-25 nM
Bisindolylmaleimide	ATP mimetic	27 nM
Imidazole [1,2-b] pyridazines	ATP competitive	40 nM
Pyridones	ATP competitive	50 nM
Flavonoids	ATP competitive	340 nM
benzoisoxazols	ATP competitive	11 nM
Isoxazoloquinoline-3,4-diones	ATP competitive	2.5 nM
5-arylidene-2,4-thiazolidinediones	ATP competitive	13 nM
Cinnamic acids	ATP competitive	11 nM
Benzo-thieno-pyrimidin-4-ones	ATP competitive	0.5 nM
Anti- Pim-1 mAb P9	Ab/Ag interaction	2.5-5 µg/ml
